# Relationship between stress hyperglycemia ratio and acute kidney injury in patients with congestive heart failure

**DOI:** 10.1186/s12933-023-02105-x

**Published:** 2024-01-13

**Authors:** Le Li, Ligang Ding, Lihui Zheng, Lingmin Wu, Zhicheng Hu, Limin Liu, Yan Yao

**Affiliations:** Peking Union Medical College, National Center for Cardiovascular Diseases, Chinese Academy of Medical Sciences, Fuwai Hospital, Beijing, 100037 China

**Keywords:** Heart failure, Acute kidney injury, Stress hyperglycemia, Restricted cubic spline

## Abstract

**Background:**

The stress hyperglycemia ratio (SHR) has been demonstrated as an independent risk factor for acute kidney injury (AKI) in certain populations. However, this relationship in patients with congestive heart failure (CHF) remains unclear. Our study sought to elucidate the relationship between SHR and AKI in patients with CHF.

**Methods:**

A total of 8268 patients with CHF were included in this study. We categorized SHR into distinct groups and evaluated its association with mortality through logistic or Cox regression analyses. Additionally, we applied the restricted cubic spline (RCS) analysis to explore the relationship between SHR as a continuous variable and the occurrence of AKI. The primary outcome of interest in this investigation was the incidence of AKI during hospitalization.

**Results:**

Within this patient cohort, a total of 5,221 (63.1%) patients experienced AKI during their hospital stay. Upon adjusting for potential confounding variables, we identified a U-shaped correlation between SHR and the occurrence of AKI, with an inflection point at 0.98. When the SHR exceeded 0.98, for each standard deviation (SD) increase, the risk of AKI was augmented by 1.32-fold (odds ratio [OR]: 1.32, 95% CI: 1.22 to 1.46). Conversely, when SHR was below 0.98, each SD decrease was associated with a pronounced increase in the risk of AKI.

**Conclusion:**

Our study reveals a U-shaped relationship between SHR and AKI in patients with CHF. Notably, we identified an inflection point at an SHR value of 0.98, signifying a critical threshold for evaluating AKI in this population.

**Supplementary Information:**

The online version contains supplementary material available at 10.1186/s12933-023-02105-x.

## Introduction

Congestive heart failure (CHF) is a leading cause of hospitalization, imposing a substantial burden on the global healthcare system [1]. CHF patients face a high mortality rate, with 30–50% succumbing or requiring rehospitalization within 60 days of admission, and a subsequent 40–50% mortality within 5 years [2]. CHF is characterized by fluid accumulation in systemic or pulmonary circulation and reduced effective circulating volume, resulting in inadequate organ perfusion. In this context, the occurrence of acute kidney injury (AKI) in CHF patients is not uncommon, with more than 40% of CHF patients manifesting coexisting renal dysfunction [3]. Notably, AKI significantly increases the risk of both in-hospital and one-year mortality by 5–7 times when compared to CHF patients without AKI [4, 5]. Recognizing CHF patients at high AKI risk early is crucial for timely interventions and vigilant care.

Stress hyperglycemia, denoting a transient increase in blood glucose levels induced by physiological or psychological stress, is frequently observed in individuals experiencing acute HF [6]. Previous studies have demonstrated that stress hyperglycemia serves as a significant risk factor of adverse outcomes [7, 8]. However, ABG levels can be influenced by chronic glycemic conditions, limiting their ability to differentiate an acute rise in blood glucose. To better gauge a patient’s actual blood glucose status, the stress hyperglycemia ratio (SHR) has been proposed [9]. It calculates the ABG while considering the individual’s average glycemic status. Several studies have suggested that SHR is highly associated with an increased risk of mortality in patients with acute HF [10], coronary artery disease [11], and stroke [12]. Furthermore, SHR exhibited a significant association with AKI in critically ill patients. Ülger’s study reported that an SHR greater than 1.47 independently served as a risk factor for AKI [13]. However, it remains uncertain whether the SHR is linked to AKI in patients with CHF. This study is designed to explore the relationship between SHR and in-hospital AKI among CHF patients admitted to critical care units.

## Methods

### Data resource

Data of this study were collected from the Medical Information Mart for Intensive Care IV (MIMIC-IV, version 2.0), a comprehensive US-based database. MIMIC-IV comprises extensive health-related data from 76,943 ICU admissions, patients received critical care at the Beth Israel Deaconess Medical Center between 2008 and 2019 [14]. The author (Le Li) had authorized access to the database with a designated Record ID of 35,965,741. To ensure patient privacy protection, all personally identifiable information has been de-identified. Since our study involved the analysis of a third-party, anonymized, publicly available database that had previously obtained institutional review board (IRB) approval, our institution’s IRB approval was considered exempt.

### Study design and participants

**Fig. 1:**
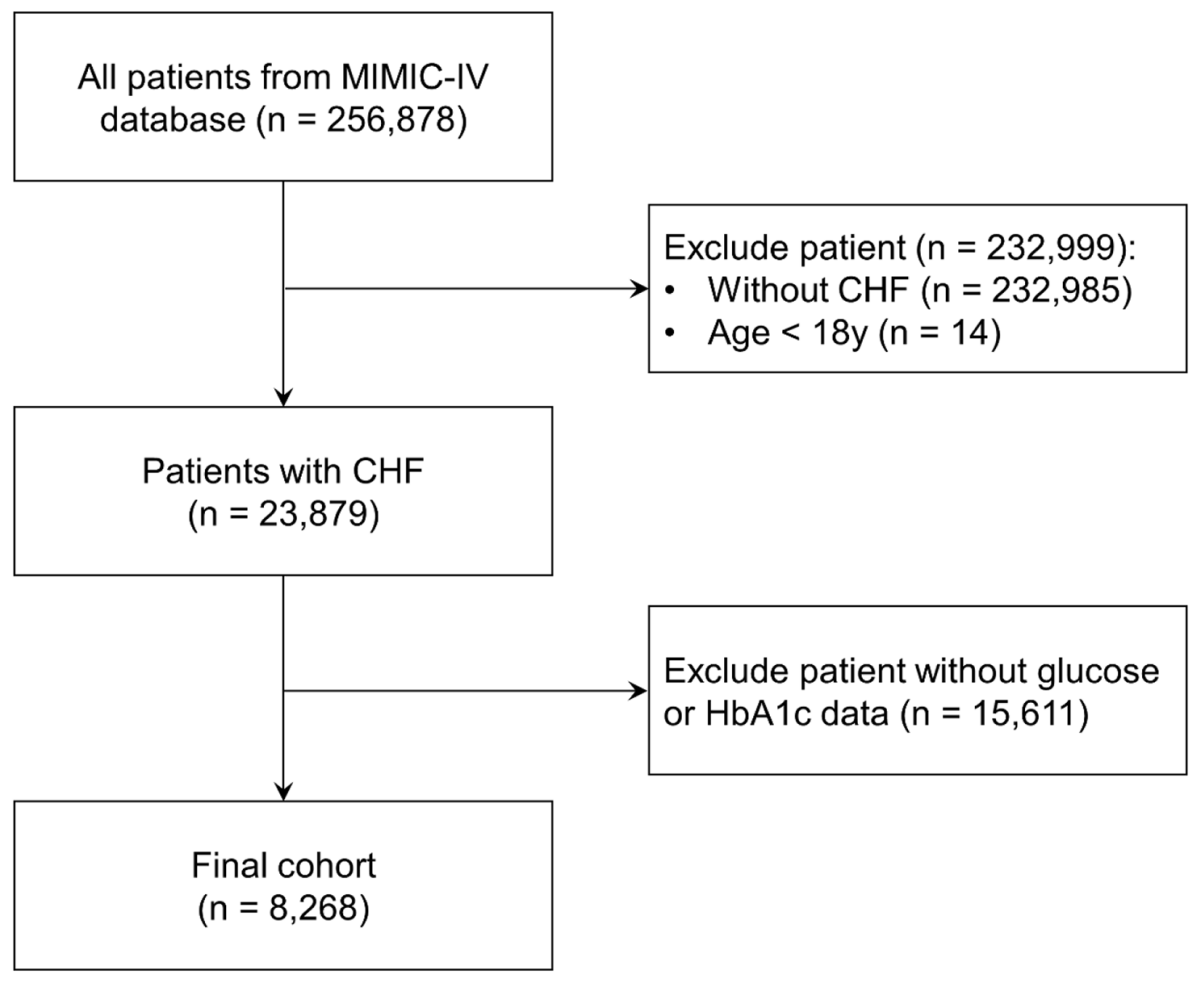
Flow chart. CHF: congestive heart failure

This is a retrospective observational cohort study based on a large-scale critical care database. Patients admitted to hospital due to CHF were included in this study. In the present study, congestive HF was diagnosed by clinicians based on the guideline of HF [15]. Based on the medical history, patients were divided into acute HF, chronic HF, and acute exacerbation in chronic HF. Patients were excluded if they were under 18 years old, without data of blood glucose or glycosylated hemoglobin A1c (HbA1c). Ultimately, a total of 8268 patients were included in the analysis (Fig. 1). Patients were stratified into seven groups based on their SHR levels, with intervals of 0.25, spanning from < 0.50 to ≥ 1.75.

### Data collection

In this study, we gathered data on patient demographics (age, sex, weight), prevalent comorbidities (including diabetes mellitus [DM], hypertension, atrial fibrillation [AF], myocardial infarction [MI], chronic kidney disease [CKD], non-ischemic cardiomyopathy [NICM], and others), laboratory parameters (such as serum creatinine [SCr], blood urea nitrogen [BUN]), medication and interventions (comprising insulin, vasopressors, diuretics, mechanical ventilation [MV], replace renal treatment [RRT]), and various pertinent variables. The diagnosis of AKI adhered to clinical practice guidelines, which include criteria such as a ≥ 0.3 mg/dL (or ≥ 26.5 µmol/L) increase in SCr within 48 h, a rise in SCr to 1.5 times the baseline level within 7 days, and a patient urine output (UO) ≤ 0.5 mL/kg/h for 6 h. Further details about AKI stage definitions can be referenced in the guidelines [16]. The SHR was calculated using the formula: SHR = ABG (mg/dL) / (28.7 × HbA1c (%)-46.7). Glucose and HbA1c values were sourced from the initial records post-ICU admission. Comorbidities were determined via ICD-9 or ICD-10 codes. Information regarding hospitalization within the first 24 h after ICU admission was meticulously extracted from the MIMIC-IV database through PostgreSQL (version 14.0). This study adhered to the Strengthening the Reporting of Observational Studies in Epidemiology (STROBE) guidelines for observational studies [17].

### Endpoints

The primary endpoint in this study was the occurrence of AKI during the hospitalization period. Secondary endpoints encompassed in-hospital mortality and one-year mortality.

### Statistical analysis

Statistical analyses were executed using R software (version 4.1.0), with statistical significance set at a two-sided *P*-value of less than 0.05. Categorical variables were represented as proportions, while continuous variables were delineated as mean (standard deviation, SD) or median (interquartile range, IQR). Continuous variables were compared using the Wilcoxon test, while categorical variables were analyzed using the chi-square test.

To explore the relationships between different levels of SHR and the probabilities of AKI and in-hospital mortality, multivariate logistic models were employed. Cox regression models were utilized to investigate the association between various SHR levels and one-year mortality. Effect sizes were quantified as odds ratios (ORs) or hazard ratios (HRs), accompanied by their respective 95% confidence intervals (CIs) for logistic or Cox models, respectively. In addition, Kaplan-Meier survival analysis was conducted to assess one-year mortality rates within SHR-defined groups, with inter-group disparities assessed using the log-rank test. Furthermore, the connection between SHR levels and mortality risk was elucidated through the application of restricted cubic spline (RCS) curves, with the reference group defined as the SHR interval demonstrating the lowest incidence rate.

The multivariate logistic and Cox regression analyses were adjusted for pertinent baseline factors, encompassing demographic parameters (age, sex), urine output, medical history (hypertension, DM, AF, acute HF, AMI, old myocardial infarction [OMI], stroke, NICM, and CKD), laboratory tests (Nt-pro BNP, SCr, BUN and HbA1c), and interventions (history of insulin use, vasopressors, loop diuretics, MV, and RRT). Furthermore, subgroup analyses were conducted to delve deeper into the data, stratifying outcomes based on age, gender, and the presence of comorbidities, such as DM, hypertension, CKD, acute HF, AMI, and NICM. These subgroup analyses were performed using comprehensive regression models adjusted for potential confounding factors.

## Results

### Baseline characteristics

A total of 8268 CHF patients were enrolled in this study, with a median age of 72.4 years (interquartile range, 62.9–81.5), and 4,665 (56.4%) were male. Among these patients, 5564 (67.3%) presented with acute heart failure. Moreover, 4687 (56.7%) were diagnosed with type 2 diabetes mellitus, and 3632 (43.9%) received insulin therapy. The participants were stratified into seven distinct groups (groups 1–7) based on their SHR levels: < 0.50 (n = 286), 0.50–0.74 (n = 1,018), 0.75–0.99 (n = 2854), 1.00-1.24 (n = 1904), 1.25–1.49 (n = 996), 1.50–1.74 (n = 530), and ≥ 1.75 (n = 680). The baseline characteristics of these seven groups are summarized in Table 1. Furthermore, for additional context, Table S1 provides a comparison of baseline characteristics between patients with AKI and those without AKI during the in-hospital period.

**Table 1:** Baseline characteristics grouped according to SHR levels

Variables	Total	Groups (group 1–7) divided by SHR							*P* value
		< 0.50	0.50–0.74	0.75–0.99	1.00–1.24	1.25–1.49	1.50–1.74	≥ 1.75	
Sample, %	8268 (100)	286 (3.5)	1018 (12.3)	2854 (34.5)	1904 (23.0)	996 (12.0)	530 (6.4)	680 (8.2)	
Age, year	72.4 (62.9–81.5)	68.1 (58.2–76.1)	72.3 (62.5–81.4)	73.3 (63.3–82.1)	72.8 (63.4–41.7)	72.9 (63.6–82.2)	72.1 (65.1–80.1)	70.5 (61.2–79.4)	< 0.001
Male, %	4665 (56.4)	163 (57.0)	579 (56.9)	1646 (57. 7)	1079 (56. 7)	535 (53.7)	281 (53.0)	382 (56.2)	0.279
Vital Signs									
SBP, mmHg	114 (106–127)	116 (106–131)	114(105–126)	114 (1056 − 125)	115 (106–126)	115(106–129)	115 (107–128)	115 (106–128)	0.121
Heart rate, bpm	82 (73–92)	81 (71–91)	81(72–91)	81 (72–90)	82 (73–92)	83 (74–93	85 (76–95)	84 (73–94)	< 0.001
Temperature, ℃	36.7 (36.5–37.0)	36.7 (36.4–36.9)	36.7 (36.5–36.9)	36.7 (36.5–37.0)	36.7 (36.5–37.0)	36.8 (36.6–37.0)	36.8 (36.6–37.0)	36.8 (36.6–37.0)	< 0.001
RR, bpm	19 (17–21)	18 (16–20)	18 (17–21)	19 (17–21)	19 (17–22)	19 (17–22)	19 (17–22)	20 (17–22)	< 0.001
Comorbidities									
Acute HF, %	5564 (67.3)	205 (71.7)	708 (69.6)	1787 (62.6)	1281 (67.3)	703 (70.6)	382 (72.1)	498 (73.2)	< 0.001
Hypertension,%	5708 (69.0)	195 (68.2)	726 (71.3)	1974 (69.2)	1323 (69.5)	668 (67.1)	369 (69.6)	453 (66.6)	0.367
AF, %	4669 (56.5)	148 (51.8)	589 (57.9)	1681 (58.9)	1078 (56.6)	537 (53.9)	295 (55.7)	341 (50.2)	< 0.001
AMI, %	1823 (22.1)	63 (22.0)	207 (20.3)	491 (17.2)	422 (22.2)	253 (25.4)	160 (30.2)	227 (33.4)	< 0.001
OMI, %	2518 (30.5)	108 (37.8)	325 (31.9)	815 (28.6)	557 (29.3)	299 (30.0)	182 (34.3)	232 (34.1)	< 0.001
NICM, %	1171 (14.16)	42 (14.7)	166 (16.3)	383 (13.4)	249 (13.1)	141 (14.2	79 (14.9)	111 (16.3)	0.129
T2DM, %	4687 (56.7)	272 (95.1)	703 (69.1)	1252 (43.9)	970 (51.0)	596 (60.0)	374 (70.6)	520 (76.5)	< 0.001
Stroke, %	2764 (33.4)	90 (31.5)	344 (33.8)	969 (33.9)	625 (32.8)	340 (34.1)	167 (31.5)	229 (33.7)	0.886
CKD, %	4211 (50.9)	228 (79.7)	598 (58.7)	1326 (46.5)	899 (47.2)	472 (47.4)	286 (54.0)	402 (59.1)	< 0.001
Laboratory tests									
NT-pro BNP, pg/mL	2482 (821–6745)	3061 (922-10085)	2465 (787–6743)	2538 (874–6712)	2372 (833–6138)	2464 (834–7020)	2424 (720–7906)	2575 (750–7428)	0.490
SCr, mg/dL	1.1 (0.8–1.5)	1.30 (0.9–2.1)	1.1 (0.9–1.6)	1.1 (0.8–1.4)	1.1 (0.8–1.4)	1.1 (0.8–1.5)	1.1 (0.9–1.6)	1.3 (0.9–1.8)	< 0.001
BUN, mg/dL	21 (15–32)	25 (17–39)	22 (15–33)	20 (15–30)	21 (15–31)	21 (15–32)	23 (16–35)	25 (17–39)	< 0.001
Glucose, mg/dL	128 (103–176)	75 (60–97)	94 (83–116)	108 (98–124)	136 (121–160)	171 (149–206)	211 (183–254)	303 (246–382)	< 0.001
HbA1c, %	6.1 (5.6-7.0)	8.6 (7.2–10.6)	6.6 (6.0–8.0)	5.9 (5.6–6.5)	5.9 (5.5–6.7)	6.0 (5.5–6.9)	6.2 (5.6–7.1)	6.4 (5.7–7.4)	< 0.001
Medical History									
Insulin use, %	3445 (41.7)	222 (77.6)	546 (53.6)	931 (32.6)	697 (36.6)	419 (42.1)	260 (49.1)	370 (54.4)	< 0.001
LD use, %	7565 (91.5)	266 (93.0)	944 (92.7)	2631 (92.2)	1728 (90.8)	905 (90.9)	482 (90.9)	609 (89.6)	0.128
Vasopressor, %	3632 (43.9)	111 (38.8)	429 (42.1)	1324 (46.4)	801 (42.1)	442 (44.4)	220 (41.5)	305 (44.9)	0.016
MV, %	6741 (81.5)	222 (77.6)	806 (79.2)	2331 (81.7)	1557 (81.8)	829 (83.2)	434 (81.9)	562 (82.7)	0.158
RRT 1st 24 h, %	491 (5.9)	45 (15.7)	71 (7.0)	125 (4.4)	102 (5.4)	50 (5.0)	40 (7.6)	58 (8.5)	< 0.001

SHR: stress hyperglycemia ratio; SBP: systolic blood pressure; RR: respiratory rate; HF: heart failure; AF: atrial fibrillation; AMI: acute myocardial infarction; OMI: old myocardial infarction; NICM: non-ischemic cardiomyopathy; T2DM: type 2 diabetes mellitus; CKD: chronic kidney injury; Nt-pro BNP: N-terminal forebrain natriuretic peptide; SCr: serum creatinine; BUN: blood urea nitrogen; HbA1c: glycosylated hemoglobin A1c; LD: loop diuretics; MV: mechanical ventilation; RRT: renal replacement treatment

### Clinical outcomes

**Fig. 2:**
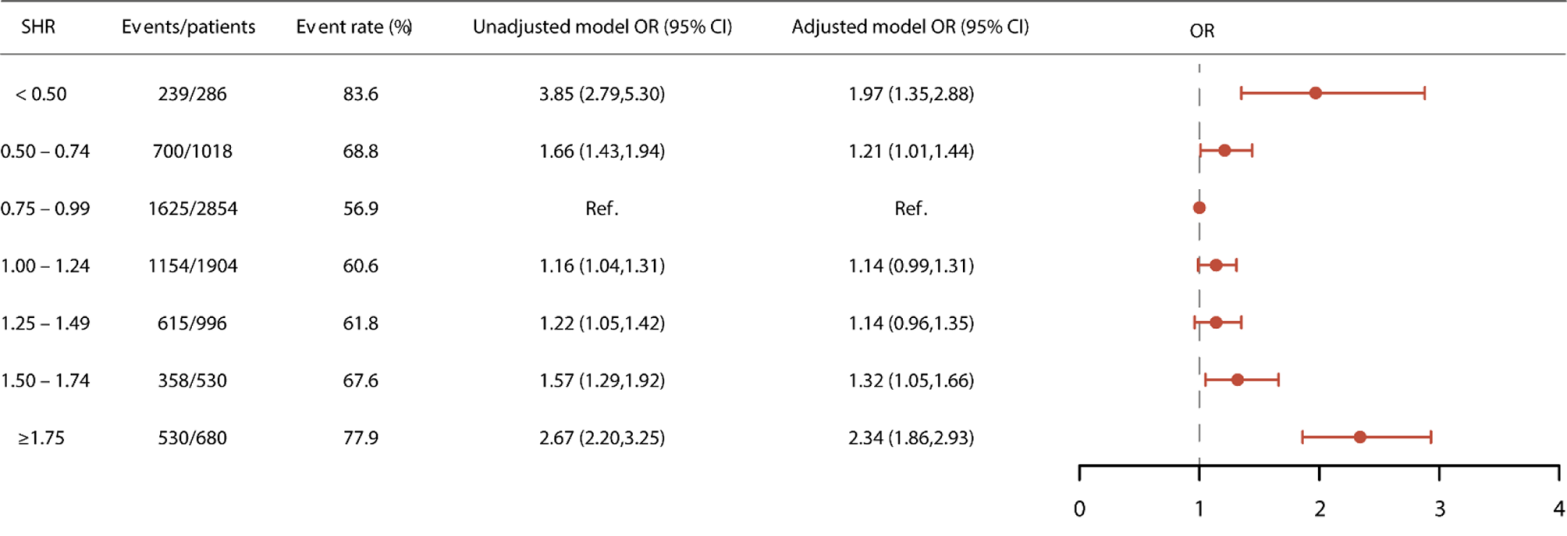
The relationship between SHR and AKI. OR: odd ratio; CI: confidence interval

**Fig. 3:**
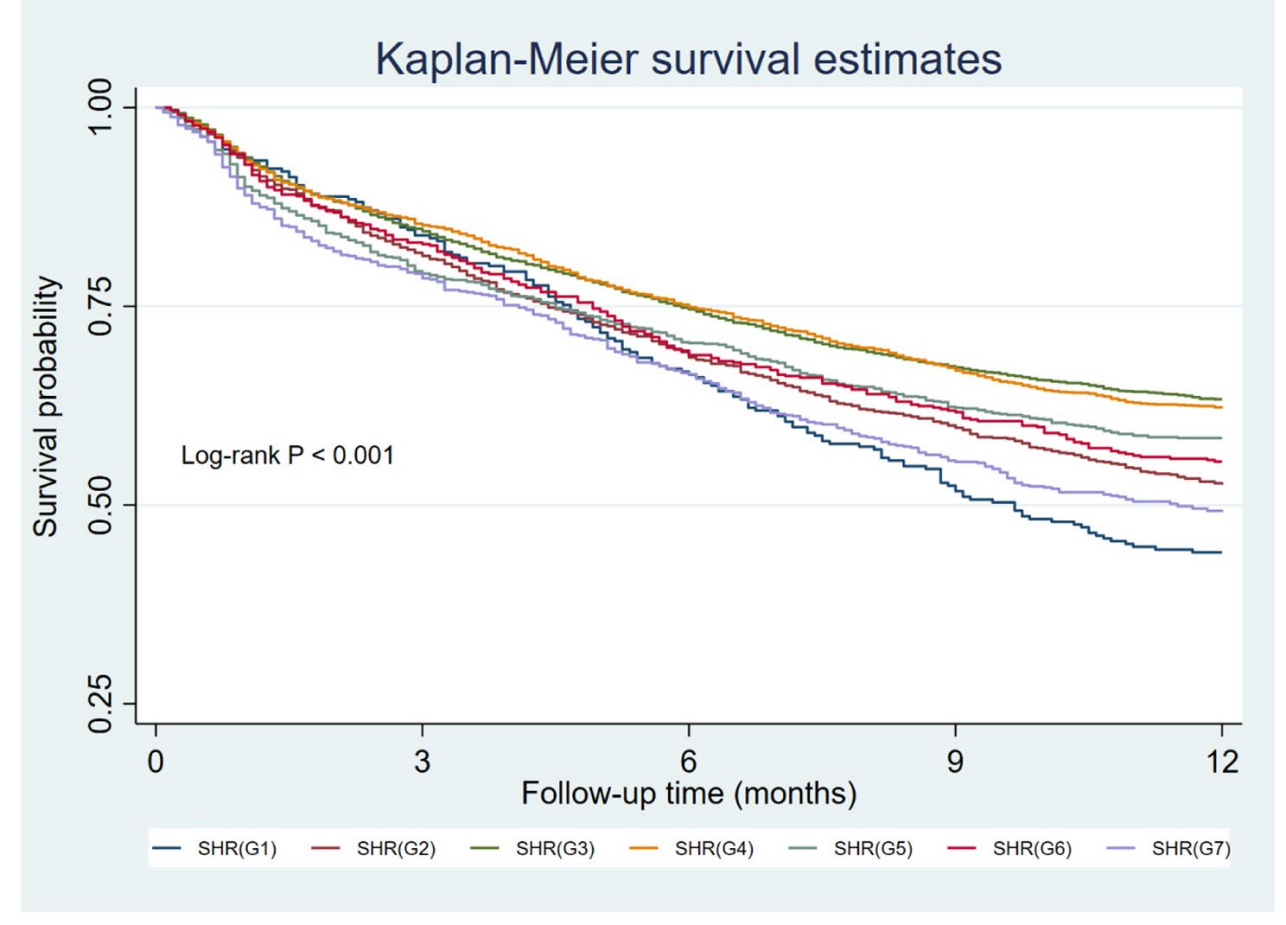
Kaplan–Meier analysis for 1-year mortality of distinct groups

In this study, AKI occurred in 5,221 (63.1%) patients during their hospitalization. Group 3 (SHR: 0.75–0.99) exhibited the lowest AKI rate at 56.9%, and was thus chosen as the reference group. In the unadjusted model, the remaining groups had a heightened risk of AKI, with ORs of 3.85 (95% CI: 2.79–5.30), 1.66 (95% CI: 1.43–1.94), 1.16 (95% CI: 1.04–1.31), 1.22 (95% CI: 1.05–1.42), 1.57 (95% CI: 1.29–1.92), and 2.67 (95% CI: 2.20–3.25) for Groups 1, 2, 4, 5, 6, and 7, respectively. This U-shaped association persisted even after adjusting for confounding variables, resulting in ORs of 1.97 (95% CI: 1.35–2.88), 1.21 (95% CI: 1.01–1.44), 1.14 (95% CI: 0.99–1.31), 1.14 (95% CI: 0.96–1.35), 1.32 (95% CI: 1.05–1.66), and 2.34 (95% CI: 1.86–2.93) for Groups 1, 2, 4, 5, 6, and 7, respectively (Fig. 2). Given the distinct management and clinical outcomes associated with AKI stage 1 and stage 2–3, we conducted logistic regressions to assess the relationship between SHR and AKI stage 2/3. Our analysis confirmed a U-shaped association between them (Figure S1). Furthermore, we observed a strong association between SHR and in-hospital mortality, with adjusted ORs of 1.56 (95% CI: 1.04 − 2.35), 1.33 (95% CI: 1.03–1.71), 1.10 (95% CI: 0.89–1.36), 1.34 (95% CI: 1.04–1.72), 1.41 (95% CI: 1.03–1.94), and 1.93 (95% CI: 1.48 − 2.52) for Groups 1, 2, 4, 5, 6, and 7, respectively. This relationship was similarly observed in the case of one-year mortality (Table 2). The Kaplan–Meier curves in Fig. 3 illustrate that patient with an SHR falling within the 0.75–0.99 range experienced the lowest one-year mortality rate (Log-rank *P* < 0.001).

**Table 2:** Cox regression and logistic regression analyses for different end points

	Events/total	Model 1			Model 2			Model 3	
		Effect size (95% CI)	*P* value		Effect size (95% CI)	*P* value		Effect size (95% CI)	*P* value
**In-hospital mortality**									
< 0.50	36/286	1.71 (1.17 − 2.48)	0.005		1.87 (1.28 − 2.73)	0.001		1.56 (1.04 − 2.35)	0.033
0.50–0.74	107/1018	1.39 (1.09–1.77)	0.007		1.42 (1.11–1.81)	0.005		1.33 (1.03–1.71)	0.029
0.75–0.99	222/2854	Ref	–		Ref	–		Ref	–
1.00–1.24	165/1904	1.12 (0.91–1.39)	0.273		1.13 (0.91–1.39)	0.272		1.10 (0.89–1.36)	0.378
1.25–1.49	103/996	1.37 (1.07–1.75)	0.013		1.37 (1.07–1.75)	0.012		1.34 (1.04–1.72)	0.024
1.50–1.74	59/530	1.49 (1.10 − 2.01)	0.011		1.52 (1.12 − 2.06)	0.007		1.41 (1.03–1.94)	0.030
≥ 1.75	100/680	2.04 (1.59 − 2.63)	< 0.001		1.87 (1.28 − 2.73)	< 0.001		1.93 (1.48 − 2.52)	< 0.001
**One-year mortality**									
< 0.50	160/286	2.19 (1.71 − 2.80)	< 0.001		2.70 (2.10–3.48)	< 0.001		1.69 (1.28 − 2.24)	< 0.001
0.50–0.74	483/1018	1.56 (1.35–1.80)	< 0.001		1.65 (1.42–1.92)	< 0.001		1.37 (1.17–1.61)	< 0.001
0.75–0.99	1047/2854	Ref	–		Ref	–		Ref	–
1.00–1.24	719/1904	1.05 (0.93–1.18)	0.451		1.05 (0.93–1.19)	0.450		1.00 (0.87–1.13)	0.946
1.25–1.49	414/996	1.23 (1.06–1.42)	0.006		1.24 (1.06–1.44)	0.006		1.16 (0.99–1.36)	0.075
1.50–1.74	236/530	1.39 (1.15–1.67)	0.001		1.45 (1.20–1.76)	< 0.001		1.24 (1.01–1.52)	0.042
≥ 1.75	346/680	1.79 (1.51 − 2.12)	< 0.001		2.00 (1.68 − 2.39)	< 0.001		1.64 (1.36–1.97)	< 0.001

Model 1: unadjusted model; Model 2: adjusted by age and sex; Model 3: adjusted by age, sex, urine output, hypertension, DM, AF, acute HF, MI, OMI, stroke, non-ischemic cardiomyopathy, CKD, Nt-pro BNP, SCr, BUN, HbA1c, history of insulin use, vasopressors, loop diuretics, MV, and RRT.

**Fig. 4:**
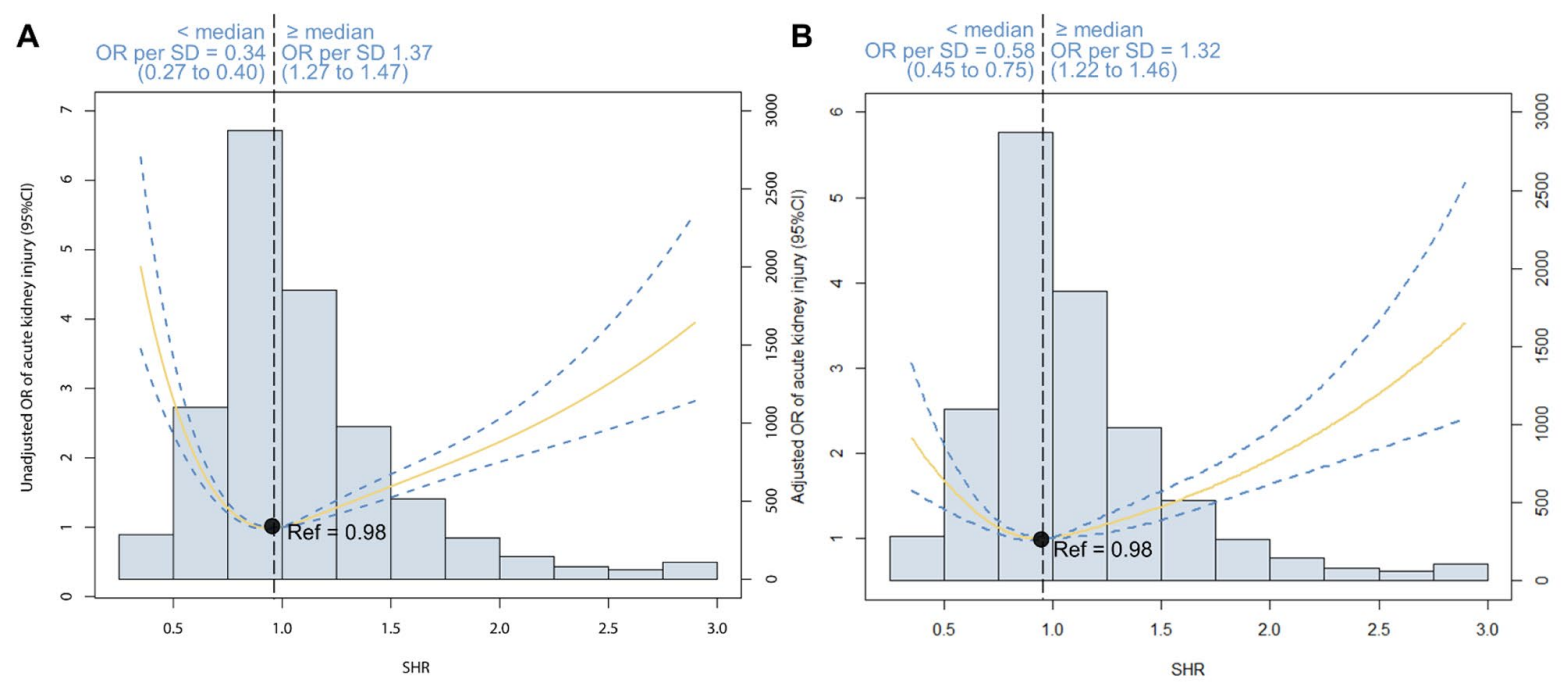
Restricted cubic spline analysis. The U-shaped association between SHR and acute kidney injury was observed in both (**A**) unadjusted model and (**B**) adjusted model

Additionally, we conducted the RCS analysis to provide a comprehensive examination of the continuous relationship between SHR and the occurrence of AKI. As depicted in Fig. 4, this graphical representation illustrates the U-shaped pattern in the association between SHR and AKI. This pattern is consistently observable in both the unadjusted and adjusted models. Notably, the optimal point in this curve, indicative of the lowest risk for all-cause mortality, was precisely identified at a specific SHR level of 0.98, aligning with the 0.75–0.99 interval (which was designated as the reference group). Upon standardizing SHR, we observed that when SHR falls below 0.98, the odds ratio for predicting AKI per standard deviation increase was 0.58 (95% CI: 0.45 to 0.75). Conversely, when SHR exceeded 0.98, the odds ratio for predicting AKI per standard deviation increase was 1.32 (95% CI: 1.22 to 1.46) based on the adjusted model. The RCS analysis of SHR and AKI stage 2/3 also illustrated the U-shaped association (Figure S2).

### Subgroup analysis

Extensive analyses were conducted to investigate the intricate association between the SHR and the development of AKI within diverse subpopulations. Rigorously adjusted for potential confounding factors, our investigation consistently revealed a recurrent U-shaped relationship across all the subgroups examined, as comprehensively presented in Table 3. Significantly, our examination unveiled notable interactions between SHR and specific variables, including gender, DM, CKD, and acute HF (all P for interaction < 0.05). For instance, it is noteworthy that non-diabetic patients exhibited a relatively elevated risk of in-hospital mortality compared to patients with CKD. Furthermore, we expanded our exploration by conducting meticulous RCS analyses within these subgroups, taking into consideration gender, DM, CKD, and acute HF. Impressively, our thorough investigation consistently disclosed a U-shaped relationship between SHR, evaluated on a continuous scale, and the incidence of AKI in all the scrutinized subgroups (Figure S3-6).

**Table 3:** Subgroup analysis evaluating the association between SHR and AKI by means of odds ratios

Variables	Groups (group 1–7) divided by SHR							P for interaction
	< 0.50	0.50–0.74	0.75–0.99	1.00–1.24	1.25–1.49	1.50–1.74	≥ 1.75	
Age								0.054
≥ 65	2.04 (1.23–3.39)	1.30 (1.05–1.62)	Ref	1.09 (0.92–1.28)	1.02 (0.83–1.25)	1.16 (0.88–1.51)	2.24 (1.68–2.97)	
< 65	1.79 (1.01–3.20)	0.97 (0.70–1.35)	Ref	1.28 (0.99–1.66)	1.52 (1.09–2.11)	1.83 (1.17–2.86)	2.49 (1.69–3.66)	
Sex								0.014
Male	2.50 (1.48–4.22)	1.23 (0.96–1.57)	Ref	1.08 (0.90–1.29)	1.16 (0.91–1.46)	1.65 (1.19–2.28)	2.97 (2.16–4.08)	
Female	1.51 (1.01–2.77)	1.18 (0.90–1.55)	Ref	1.22 (0.99–1.51)	1.11 (0.86–1.43)	1.07 (0.77–1.48)	1.75 (1.26–2.43)	
DM								< 0.001
Yes	1.74 (1.17–2.60)	1.16 (0.91–1.47)	Ref	1.02 (0.83–1.25)	0.92 (0.72–1.16)	1.23 (0.91–1.65)	1.81 (1.38–2.40)	
No	3.05 (0.80–11.7)	1.14 (0.86–1.52)	Ref	1.23 (1.02–1.49)	1.42 (1.10–1.82)	1.37 (0.94–2.01)	3.49 (2.33–5.23)	
Hypertension								0.152
Yes	2.22 (1.32–3.74)	1.37 (1.10–1.71)	Ref	1.22 (1.03–1.44)	1.20 (0.97–1.49)	1.36 (1.02–1.80)	2.05 (1.54–2.71)	
No	1.72 (0.98–3.01)	0.95 (0.70–1.30)	Ref	1.00 (0.79–1.28)	1.04 (0.78–1.40)	1.22 (0.82–1.81)	2.82 (1.94–4.13)	
CKD								0.002
Yes	1.88 (1.11–3.18)	1.06 (0.80–1.42)	Ref	0.94 (0.75–1.20)	1.15 (0.85–1.56)	1.33 (0.89–1.98)	1.71 (1.19–2.47)	
No	1.99 (1.12–3.54)	1.29 (1.02–1.62)	Ref	1.25 (1.05–1.48)	1.14 (0.92–1.41)	1.27 (0.95–1.69)	2.60 (1.94–3.41)	
Acute HF								0.017
Yes	2.11 (1.25–3.58)	1.37 (1.09–1.74)	Ref	1.14 (0.96–1.36)	1.22 (0.98–1.51)	1.28 (0.96–1.71)	2.06 (1.55–2.73)	
No	1.82 (1.04–3.17)	1.02 (0.77–1.36)	Ref	1.14 (0.92–1.42)	1.02 (0.76–1.36)	1.33 (0.91–1.95)	3.10 (2.11–4.54)	
AMI								0.429
Yes	1.47 (0.62–3.49)	1.00 (0.64–1.57)	Ref	0.91 (0.66–1.26)	0.76 (0.53–1.10)	1.29 (0.81–2.06)	1.73 (1.13–2.65)	
No	2.07 (1.36–3.16)	1.25 (1.02–1.52)	Ref	1.20 (1.03–1.39)	1.26 (1.04–1.54)	1.27 (0.97–1.65)	2.52 (1.92–3.31)	
NICM								0.312
Yes	2.41 (0.69–8.47)	1.26 (0.75–2.13)	Ref	1.11 (0.73–1.67)	0.95 (0.58–1.57)	1.07 (0.54–2.12)	1.66 (0.88–3.13)	
No	1.92 (1.29–2.85)	1.18 (0.98–1.43)	Ref	1.14 (0.98–1.32)	1.16 (0.96–1.39)	1.35 (1.06–1.72)	2.43 (1.91–3.10)	

The abbreviations are as same in Table 1

## Discussion

To the best of our knowledge, this study marks the first comprehensive investigation into the association between SHR and AKI in patients with CHF. Our findings have revealed a distinctive U-shaped correlation between SHR and the occurrence of AKI within this patient cohort. Specifically, both elevated and reduced SHR values demonstrate a significant connection with an increased susceptibility to AKI during hospitalization, with a turning point for SHR at 0.98. Furthermore, our research has illuminated SHR as a potential risk factor for all-cause mortality in this particular patient population.

The concept of SHR underscores a relative acute increase in glycemia when compared to the individual’s previous glycemic status in response to a stress reaction or critical illness [9]. Recently, we have demonstrated the strong association of SHR and all-cause mortality in patients with critical illness [18]. In the study, we enrolled 8,978 patients with critical illness and identified a U-shaped relationship between SHR and mortality. Notably, the SHR falling within the range of 0.75 to 0.99 exhibited the lowest mortality rates. For each increment of 0.25 in the SHR within this range, the risk of in-hospital mortality increased by 1.34-fold (OR: 1.34, 95% CI: 1.25–1.44). Conversely, a decrease of 0.25 in SHR within the same 0.75–0.99 range elevated the risk by 1.38-fold (OR: 1.38, 95% CI: 1.10–1.75). In addition, Wei et al. suggested that SHR was significantly associated with an increased risk of in-hospital death and all-cause mortality in ST-element myocardial infarction patients treated with percutaneous coronary intervention [19]. In this study, we also found that SHR was highly associated with all-cause mortality in CHF patients. However, the relationship between SHR and AKI in this population remains unclear.

CHF is characterized by the presence of congestion within the systemic or pulmonary circulation, which results in a reduction in effective circulating volume, ultimately leading to inadequate organ perfusion. Conversely, a decline in the glomerular filtration rate, as seen in AKI, augments the cardiac volume load and exacerbates cardiac dysfunction. These intricate and reciprocal interactions between the kidney and heart are collectively referred to as cardiorenal syndrome [20]. Multiple studies have provided evidence that acute hyperglycemia independently predicts the occurrence of AKI in specific patient populations [21, 22]. Recently, Gao et al. suggested that the novel index SHR serves as a superior predictor of AKI compared to ABG in a cohort of 1,215 diabetic patients with AMI [23]. In this study, we have firstly provided the report highlighting a robust association between the SHR and the occurrence of AKI in patients with CHF. This association, marked by a U-shaped pattern, not only emphasizes critical clinical implications but also provides vital insights for optimizing personalized risk assessment strategies, directing targeted interventions, and laying the groundwork for future research to unravel underlying mechanisms. The underlying mechanisms of the U-shaped association remains unclear. Some findings may help to explain the mechanisms. Firstly, stress hyperglycemia is driven by the hypothalamic-pituitary-adrenal axis and the sympathoadrenal system to reestablish homeostasis amidst intense stress [24]. Some studies suggested that mild-to-moderate stress hyperglycemia is a protective factor during stress [24]. McNamara et al. have discovered that stress hyperglycemia may enhance cardiac output and contribute to improved survival [25]. Additionally, stress hyperglycemia has the capacity to increase the expression of cell survival factors, notably vascular endothelial growth factor and hypoxia-inducible factor-1α. Consequently, this results in a decrease in cellular apoptosis, a reduction in infarction size, and improvements in cardiac systolic function within a rat model of MI [26]. Furthermore, stress hyperglycemia leads to the establishment of a new glucose equilibrium, creating a heightened blood glucose diffusion gradient that optimizes cellular glucose uptake, particularly in the presence of microvascular flow disturbances. Consequently, moderate hyperglycemia maximizes cellular glucose uptake while mitigating the risk of hyperosmolarity [27].

Furthermore, we identified the inflection point of SHR for poor prognosis to be 0.98. Consequently, an SHR greater than 0.98 is indicative of stress hyperglycemia. In contrast, SHR values below 0.98 suggest the presence of chronic hyperglycemia (as indicated by high HbA1c) with either effective current glycemic control or the potential for glycemic control that exceeds the target levels set by ABG. This U-shaped association suggested that the risk increases in all situations that depart from the linear correlation between ABG and HbA1c in both directions.

### Limitations

While we have provided the initial evidence of a U-shaped association between SHR and AKI in CHF patients, it is essential to acknowledge certain limitations in our study. Firstly, this was a retrospective observational study conducted at a single center, which precludes us from establishing a definitive causal association between this factor and clinical outcomes. Secondly, the data for this study were derived from a third-party public database, which led to the exclusion of certain covariates, including left ventricular ejection fraction, from the multivariable regression models. Thirdly, while our primary focus was on the CHF population, it is worth noting that the patients in this cohort were primarily hospitalized for critical illness, which may limit the generalizability of our findings to the broader population. Therefore, the findings of this study should be interpreted with caution.

## Conclusion

In conclusion, our study has unveiled a U-shaped association between SHR and AKI in patients with CHF. Notably, both low and high SHR values exhibited significant associations with AKI and mortality in this specific patient population, underscoring the paramount importance of maintaining optimal glycemic control in this clinical context. Furthermore, the inflection point for SHR concerning AKI was precisely identified at 0.98, marking a critical threshold for risk assessment and intervention. Future research endeavors should aim for larger-scale, multicenter, and prospective investigations to further substantiate our findings.

### Electronic supplementary material

Below is the link to the electronic supplementary material.

Supplementary Material 1

## Data Availability

The datasets used during the current study are available from the corresponding author on reasonable request.
