# Supplementary material for: Relationship between stress hyperglycemia ratio and acute kidney injury in patients with congestive heart failure
Source: Cardiovasc Diabetol. 2024 Jan 13;23:29. doi: 10.1186/s12933-023-02105-x (PMC10787441; doi:10.1186/s12933-023-02105-x)
Supplement: Supplementary file 1 — Supplementary Material 1 [file 12933_2023_2105_MOESM1_ESM.docx]

| **Table S1**. Baseline characteristics grouped by acute kidney injury status. | | | | |
| --- | --- | --- | --- | --- |
| **Variable** | **Total**  **(n = 8268)** | **Non-AKI**  **(n = 3047)** | **AKI**  **(n = 5221)** | **P value** |
| Age, year | 72.4 (62.9 - 81.5) | 71.4 (61.8 - 80.7) | 73.1 (63.4 - 81.9) | <.001 |
| Male, % | 4665 (56.4) | 1682 (55.2) | 2983 (57.1) | 0.087 |
| Vital Signs |  |  |  |  |
| SBP, mmHg | 114 (106 - 127) | 114 (106 - 125) | 115 (105 - 128) | 0.125 |
| Heart rate, bpm | 82 (73 - 92) | 82 (74 - 90) | 82 (72 - 93) | 0.528 |
| Temperature, ℃ | 36.7 (36.5 - 37.0) | 36.8 (36.6 - 37.0) | 36.7 (36.5 - 37.0) | 0.010 |
| RR, bpm | 19 (17 - 21) | 18 (17 - 21) | 19 (17 - 22) | <.001 |
| Comorbidities |  |  |  |  |
| Acute HF, % | 5564 (67.3) | 1588 (52.1) | 3976 (76.2) | <.001 |
| Hypertension, % | 5708 (69.0) | 1933 (63.4) | 3775 (72.3) | <.001 |
| AF, % | 4669 (56.5) | 1596 (52.4) | 3073 (58.9) | <.001 |
| AMI, % | 1823 (22.1) | 552 (18.1) | 1271 (24.3) | <.001 |
| OMI, % | 2518 (30.5) | 769 (25.2) | 1749 (33.5) | <.001 |
| NICM, % | 1171 (14.16) | 309 (10.1) | 862 (16.5) | <.001 |
| T2DM, % | 4687 (56.7) | 1362 (44.7) | 3325 (63.7) | <.001 |
| Stroke, % | 2764 (33.4) | 918 (30.1) | 1846 (35.4) | <.001 |
| CKD, % | 4211 (50.9) | 706 (23.2) | 3505 (67.1) | <.001 |
| Laboratory tests |  |  |  |  |
| NT-pro BNP, pg/mL | 2482 (821 - 6745) | 2482 (821 - 6745) | 2049 (664 - 5109) | <.001 |
| SCr, mg/dL | 1.1 (0.8 - 1.5) | 0.9 (0.7 - 1.2) | 1.2 (0.9 - 1.7) | <.001 |
| BUN, mg/dL | 21 (15 - 32) | 18 (14- 25) | 24 (17 - 37) | <.001 |
| Glucose, mg/dL | 128 (103-176) | 123 (101-162) | 132 (105-185) | <.001 |
| HbA1c, % | 6.1 (5.6-7.0) | 5.9 (5.5-6.6) | 6.2 (5.7-7.2) | <.001 |
| Medical History |  |  |  |  |
| Insulin use, % | 3445 (41.7) | 739 (24.3) | 2706 (51.8) | <.001 |
| LD use, % | 7565 (91.5) | 2632 (86.4) | 4933 (94.5) | <.001 |
| Vasopressor, % | 3632 (43.9) | 1471 (48.3) | 2161 (41.4) | <.001 |
| MV, % | 6741 (81.5) | 2561 (84.1) | 4180 (80.1) | <.001 |
| RRT 1st 24h, % | 491 (5.9) | 155 (5.1) | 336 (6.4) | 0.012 |

The abbreviations are as same in Table 1.


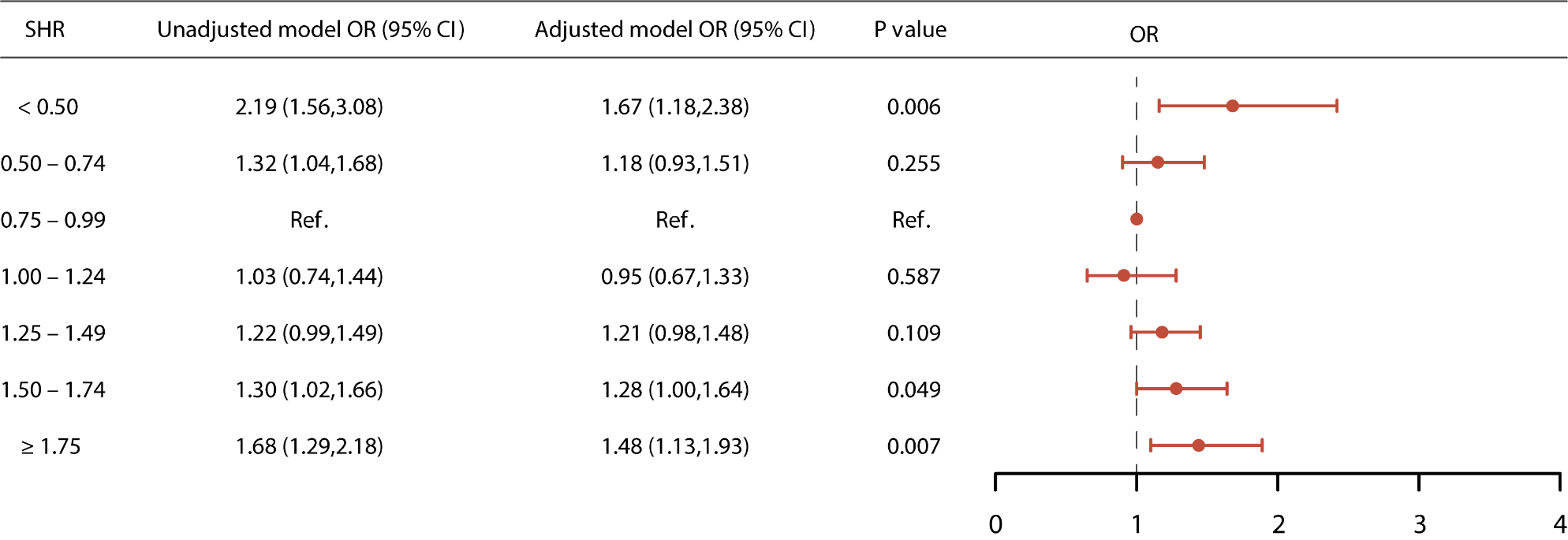


**Figure 2**. The relationship between SHR and AKI stage 2/3. OR: odd ratio; CI: confidence interval.


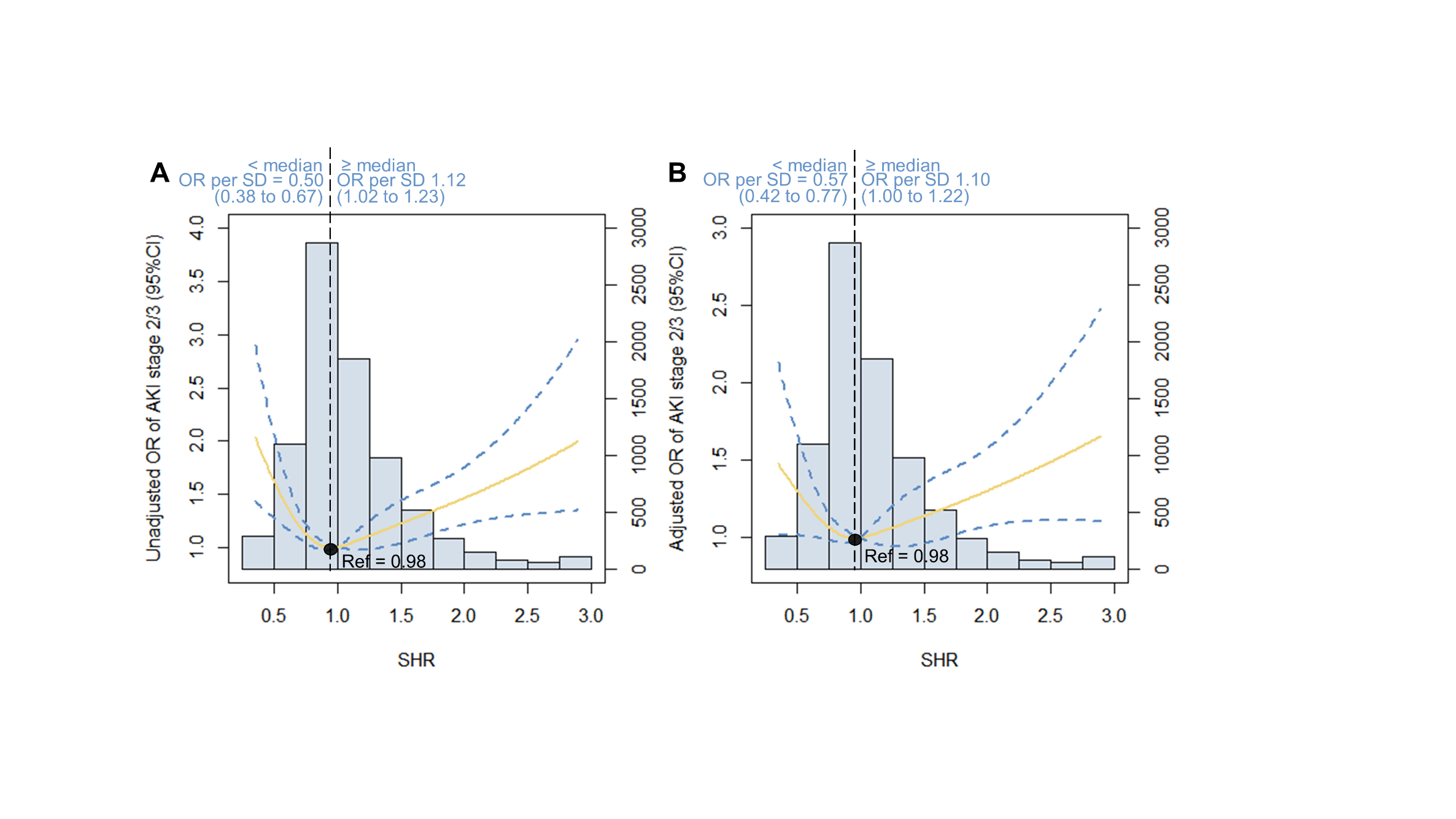


**Figure S1**. Association between SHR and AKI stage 2/3 depicted by restricted cubic spline curve in unadjusted (**A**) and adjusted (**B**) models.


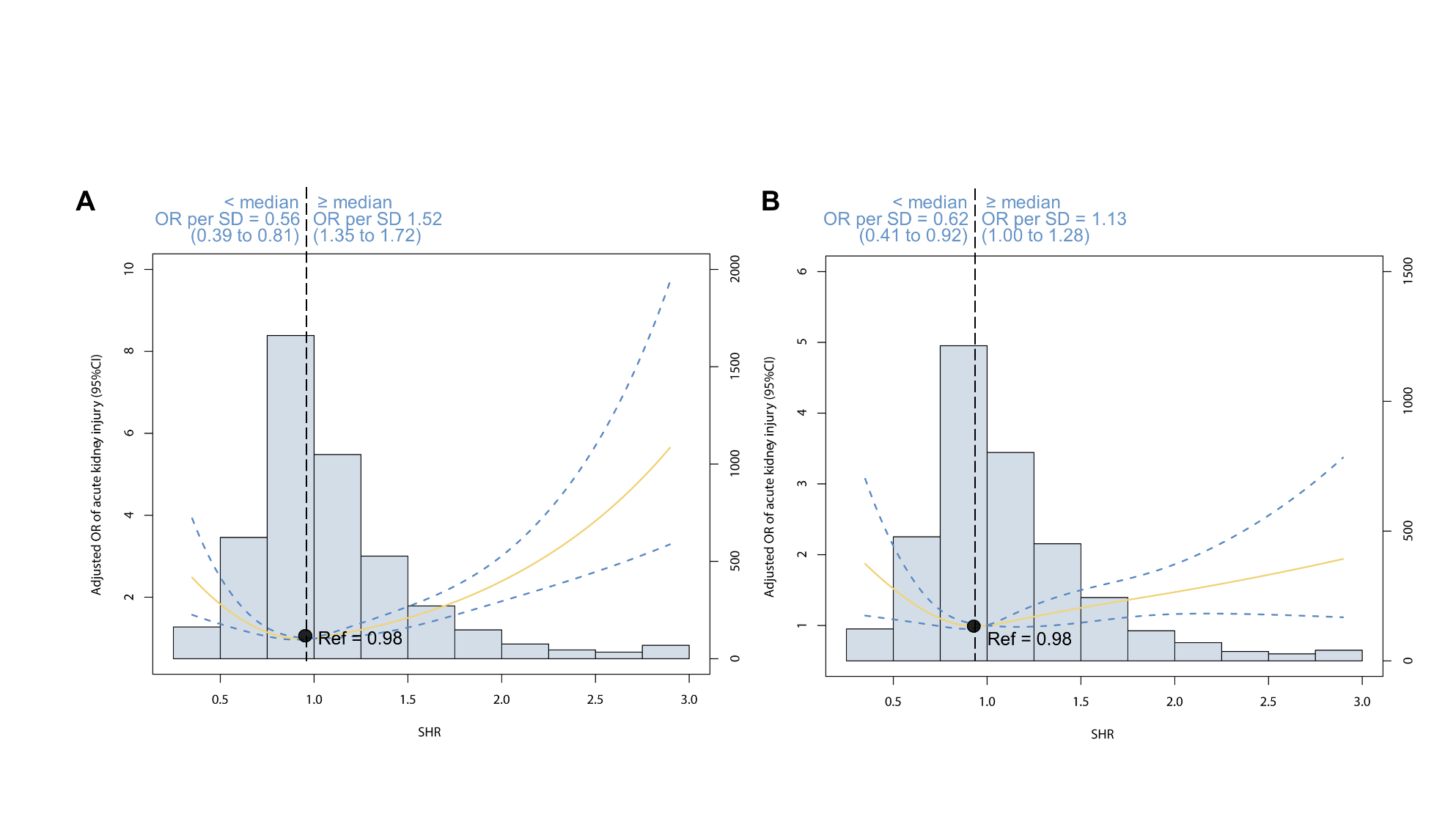


**Figure S2**. Association between SHR and AKI depicted by restricted cubic spline curve in male (**A**) and female (**B**).


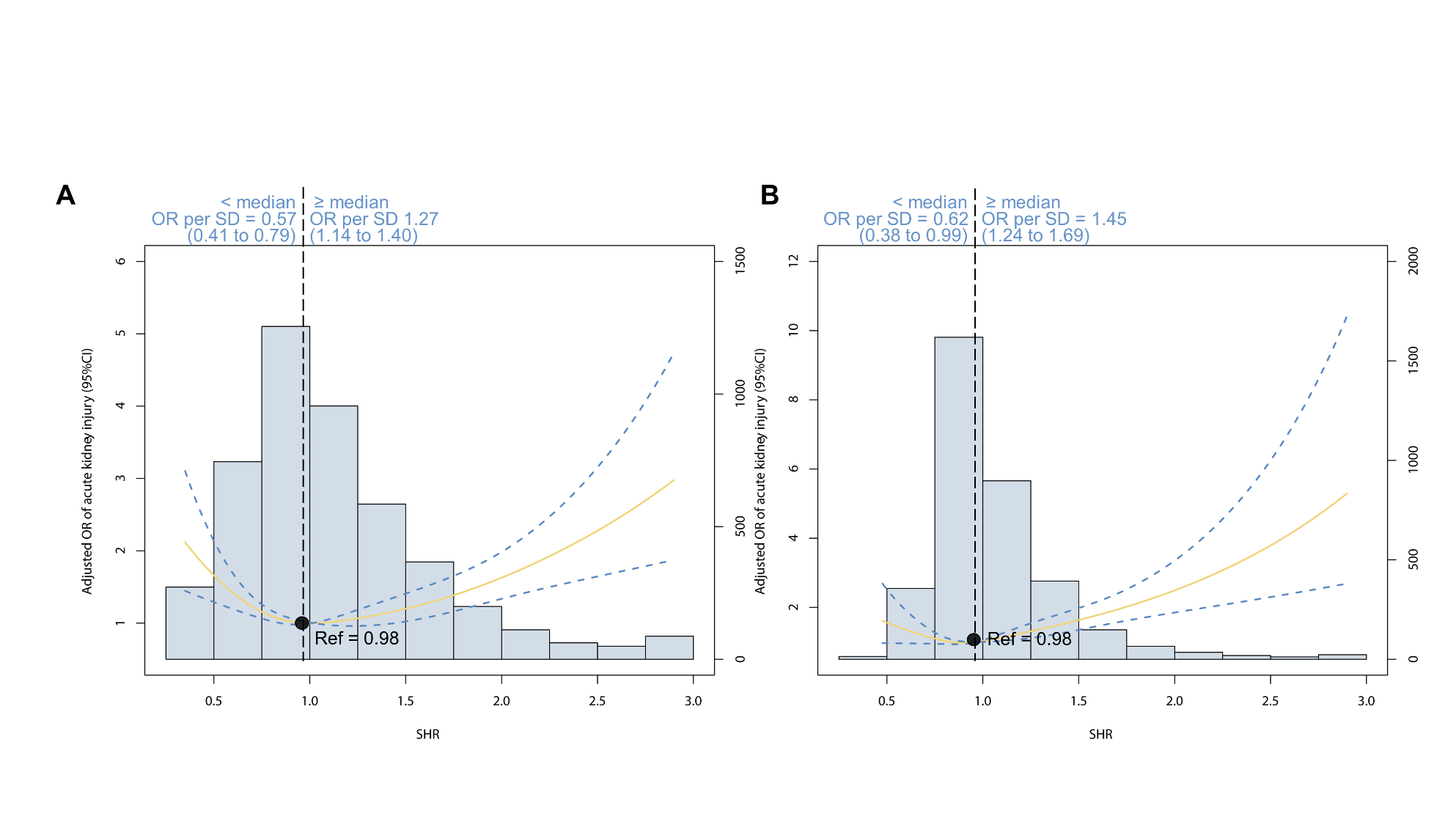


**Figure S3**. Association between SHR and AKI depicted by restricted cubic spline curve in patients with (**A**) or without (**B**) diabetes mellitus.


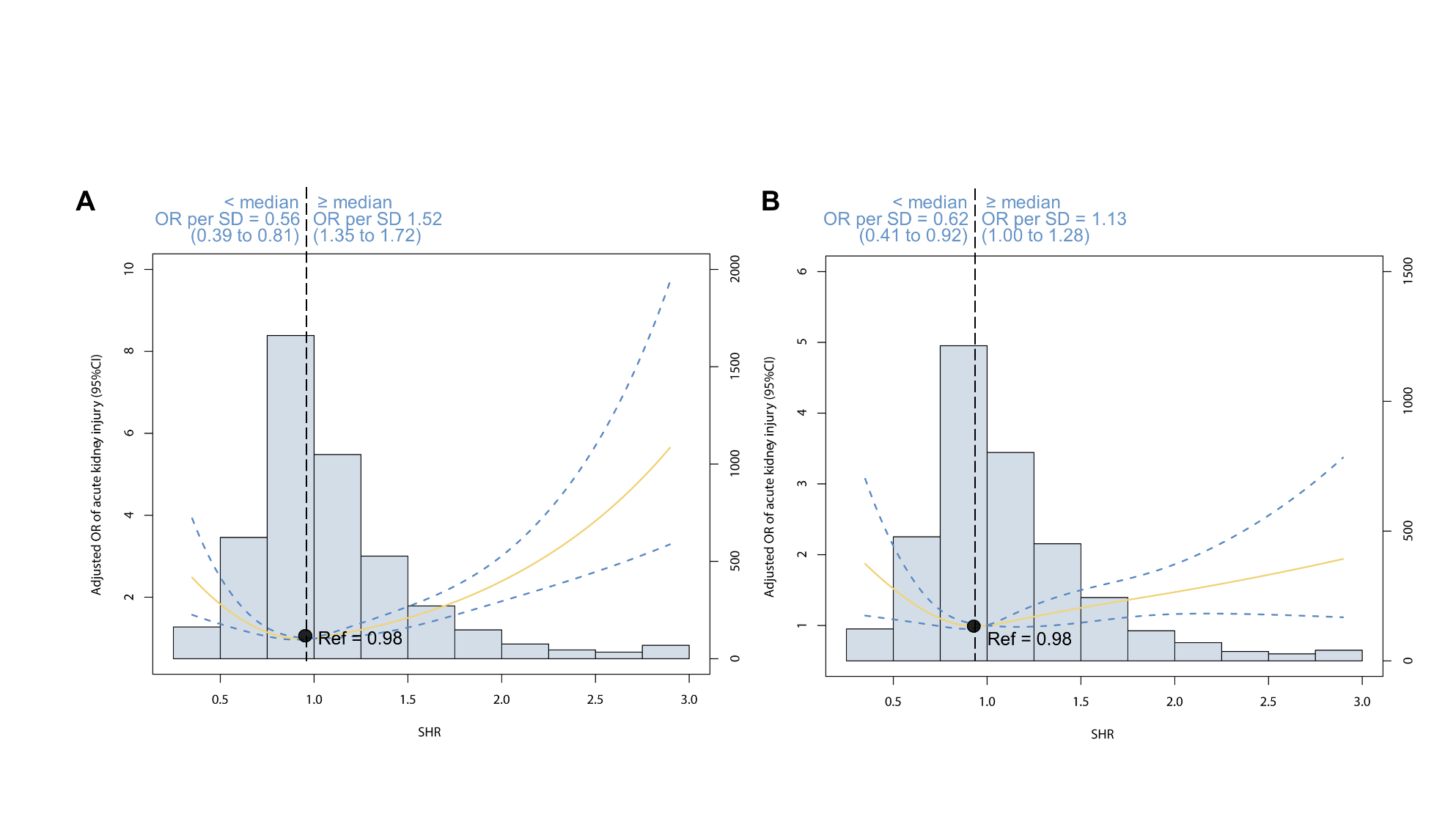


**Figure S4**. Association between SHR and AKI depicted by restricted cubic spline curve in patients with (**A**) or without (**B**) chronic kidney disease.


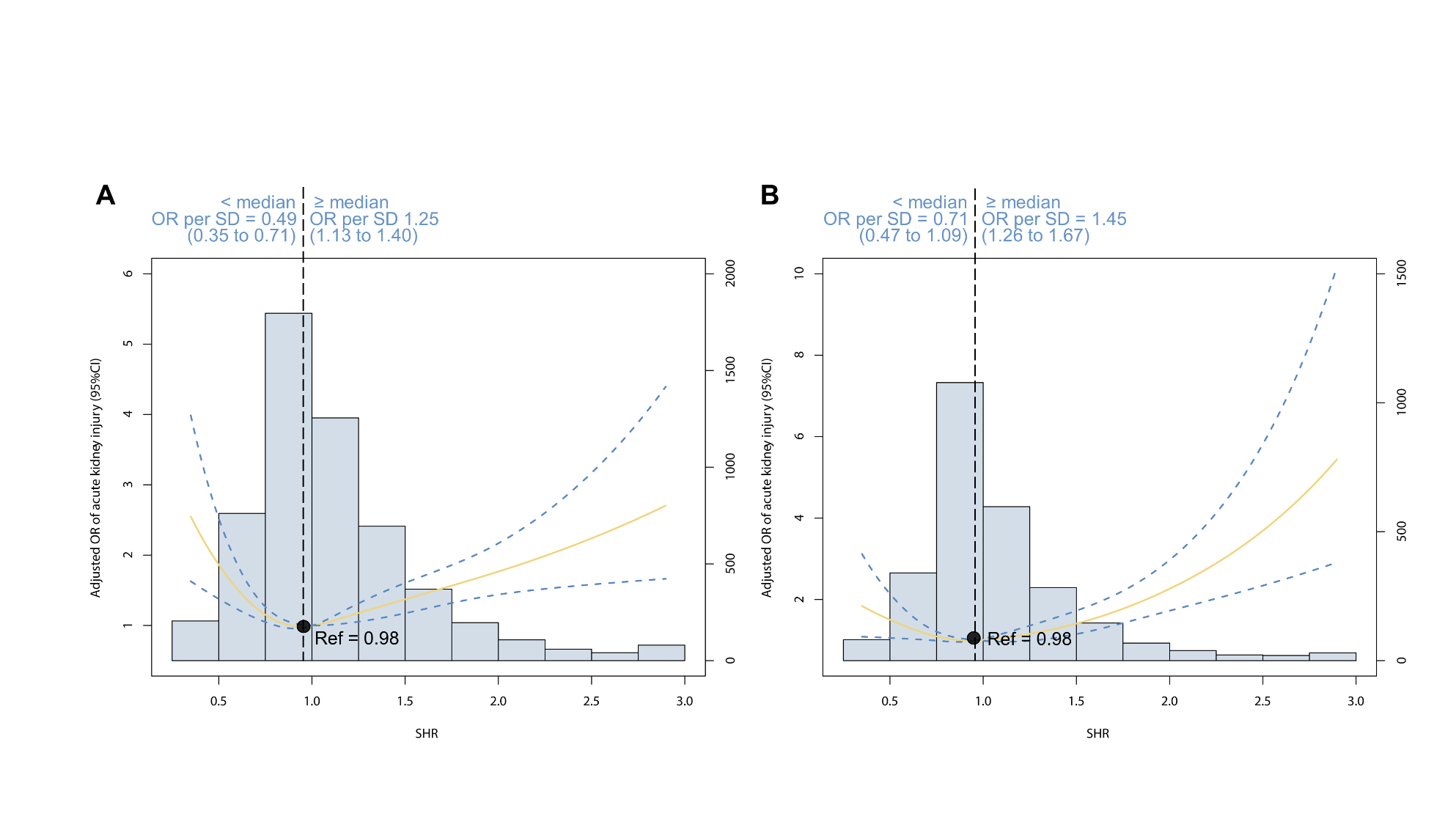


**Figure S5**. Association between SHR and AKI depicted by restricted cubic spline curve in patients with (**A**) or without (**B**) acute heart failure.
